# Challenging Management of a Patient With Severe Bilateral Deep Vein Thrombosis

**DOI:** 10.1177/2324709620910288

**Published:** 2020-03-05

**Authors:** Mohanad Hamandi, Allison T. Lanfear, Seth Woolbert, Madison L. Bolin, Joy Fan, Michael William, Zoheb Khan, J. Michael DiMaio, Chadi Dib

**Affiliations:** 1 Baylor Scott and White—The Heart Hospital, Plano, TX, USA

**Keywords:** DVT, thrombectomy, thrombolysis

## Abstract

Among patients with proximal iliofemoral deep vein thrombosis (DVT) and an elevated Villalta score, anticoagulation therapy alone may not be a sufficient management strategy in select cases. In this article, we report a case of severe bilateral iliofemoral DVT that resisted the standard treatment for DVT, requiring catheter-directed thrombolysis and subsequent mechanical thrombectomy.

## Introduction

Venous thromboembolism (VTE) affects 1 in 500 people annually in the United States, with a 13% mortality rate primarily due to pulmonary embolism (PE).^1,2^ Proximal deep vein thrombosis (DVT) mainly involving the femoral and iliac veins carries a much higher risk of PE and symptomatic PE when compared with distal DVT.^3^ Iliofemoral DVT accounts for 39% of all proximal DVT cases and carries a higher risk of recurrent VTE.^3,4^ One third of patients diagnosed with DVT/PE have a recurrence within 10 years, and up to 50% develop post-thrombotic syndrome (PTS).^5^ Anticoagulation therapy is the standard treatment for VTE, as it reduces thrombus extension, recurrence, and the risk of PTS.^2^ Inferior vena cava (IVC) filters, thrombolysis, surgical thrombus removal, and compression stockings are also available treatment options.^2^ In this article, we report a case of severe bilateral iliofemoral DVT that resisted the standard treatment for DVT and required catheter-directed thrombolysis (CDT) and subsequent mechanical thrombectomy.

## Case Summary

**Figure 1.**
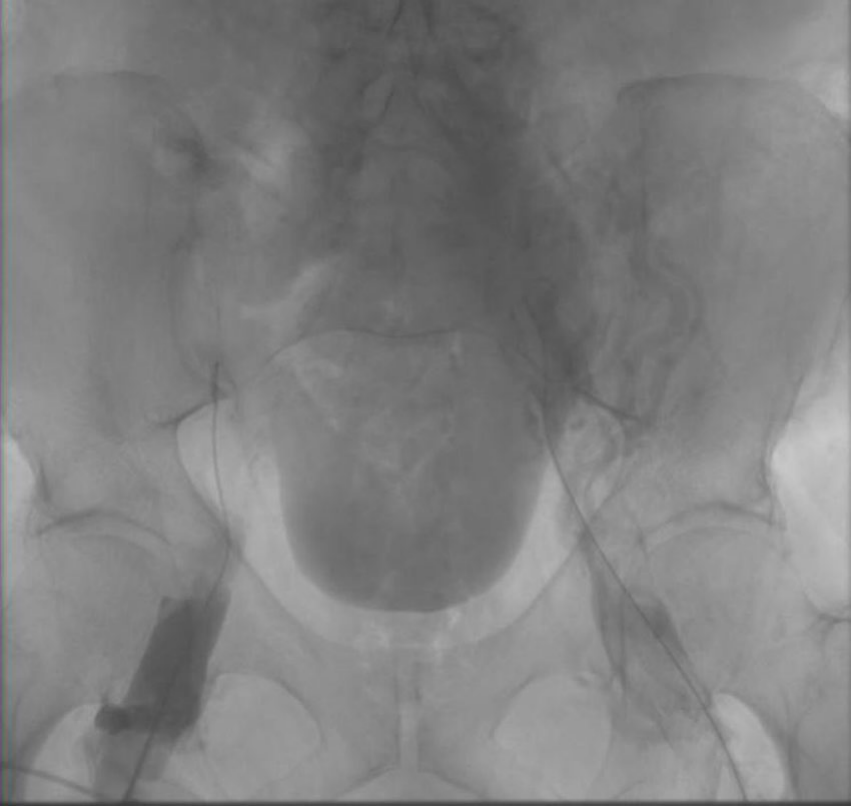
Venogram of bilateral femoral and iliac veins on admission.

**Figure 2.**
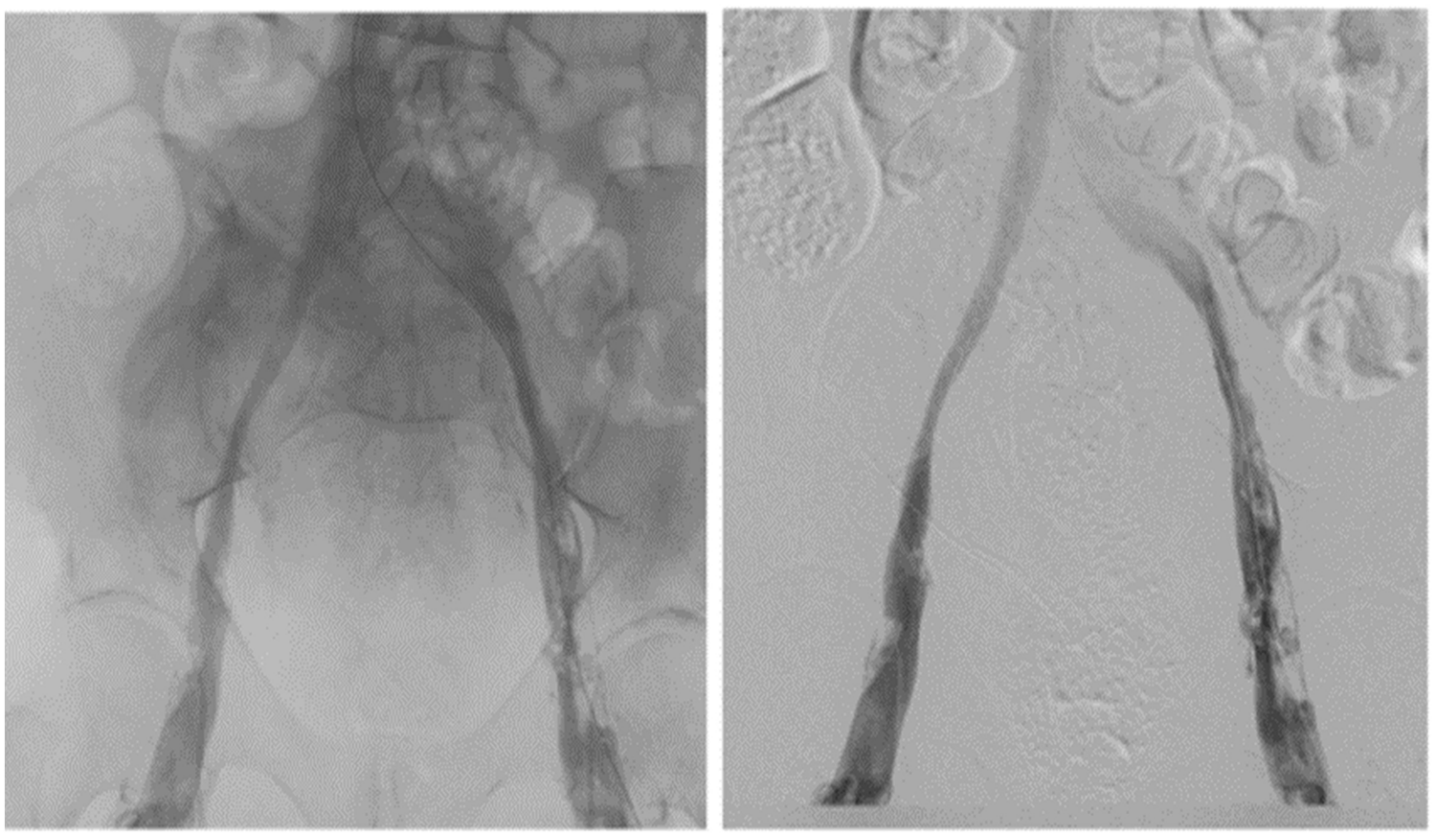
Venogram of bilateral femoral and iliac veins after treatment with EKOS.

**Figure 3.**
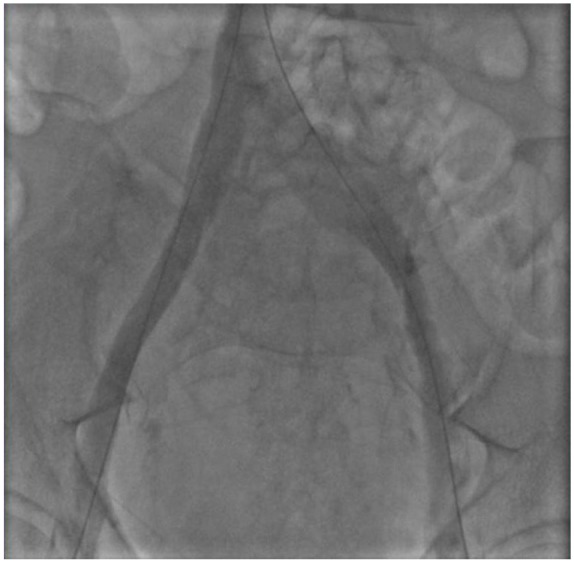
Venogram of bilateral femoral and iliac veins after treatment with Penumbra.

A 56-year-old man with a history of hemochromatosis and noncompliance was referred to the emergency room for bilateral lower extremity (LE) swelling, pain, weeping lesions, and difficulty walking. One month prior, the patient was diagnosed with bilateral DVT to the superficial femoral veins and managed with oral anticoagulation therapy (rivaroxaban). An IVC filter was implanted due to his history of recurrent LE DVT, lack of response to direct oral anticoagulants, and large clot burden at an outside hospital. He also has a history of smoking, peripheral vascular disease, diabetes, congestive heart failure, and morbid obesity with a body mass index of 46.1 kg/m^2^. He denied any history of coagulopathies or recent travel. Color and pulsed Doppler sonography of the bilateral LE deep venous system and distal compression for flow augmentation were performed. Homogenous, hypoechoic, low-level internal echoes filled the lumen of the right and the left common femoral veins, extended into the right deep femoral vein, and prevented complete compression. No flow was seen on color and pulsed Doppler evaluation. The superficial femoral, popliteal, posterior tibial, and peroneal veins were widely patent. Computed tomography angiography of the chest showed linear nonocclusive filling defects in the second- and third-order pulmonary arteries supplying the right lung consistent with pulmonary emboli, likely chronic. The patient reported noncompliance with rivaroxaban, and on admission to our hospital, his international normalized ratio was found to be 1.5. Rivaroxaban was stopped, and he was started on enoxaparin. Enoxaparin was adjusted by 1 mg/kg subcutaneous twice a day.^6^ His Villalta score was 26. Following examination and review of the case with our integrated PERT (pulmonary embolism response team)/DVT team, we decided to proceed with intervention. We accessed the right and left distal femoral veins and advanced two 6 Fr sheaths. Venography showed complete occlusion of the proximal femoral veins bilaterally, external, and common iliac veins along with occlusion of the distal IVC up the filter (Figure 1). We then introduced 2 EKOS (EndoWave Infusion Catheter System; EKOS Corp, Bothell, WA) catheters in each access site delivering tPA with ultrasonic frequency from the right common femoral vein to the IVC and from the left common femoral vein to the proximal left common iliac vein. After 2 days of therapy, a venogram showed reestablishment of flow in both femoral, external, and common iliac veins up to the IVC (Figure 2). However, a large amount of clot was noted, mostly located in the right and left common femoral veins and left external and common iliac vein. Mechanical thrombectomy was then performed using an 8 Fr Penumbra Indigo catheter in both the right and left lower extremities. Subsequently, there were no filling defects seen angiographically (Figure 3). The patient was discharged on subcutaneous enoxaparin for 3 months to be followed by warfarin with a goal international normalized ratio of 2 to 3. The patient was also instructed to wear thigh-high 20 to 30 mm Hg gradual compression socks on bilateral LE for life. Continuous education and phone calls were made to ensure compliance with medications. At 3-month follow-up, the patient was doing well; his LE swelling, although present, was not life-limiting, and his Villalta score was down to 13.

## Discussion

DVT is typically found in the LE and seen in 0.1% of people annually.^7^ This patient’s history of hemochromatosis and increased blood viscosity with a hemoglobin of 19 likely augmented his risk for acute on chronic DVT. His body mass index of 46.1 kg/m^2^ is also estimated to increase the risk of DVT by a factor of 2.^8,9^ Furthermore, his low hemodynamic flow rate caused by his congestive heart failure and the presence of an IVC filter may have increased his predisposition for DVT.^1,9^

Anticoagulation has been the standard treatment approach for DVT; however, in patients with iliofemoral DVT and thrombosis extending into the IVC, the choice of therapy remains debatable. It is well established that iliofemoral DVT results in more severe post-thrombotic venous hypertension, more frequent recurrent DVT, and more frequent and severe PTS.^10^ Although it is difficult to predict PTS occurrence in patients with DVT, treatment that provides rapid resolution of the venous obstruction is essential to avoid diminished quality of life.

To diagnose and categorize the severity of PTS, the Villalta scoring system is often used. This score uses a point system that tracks 5 symptoms (pain, cramps, heaviness, paresthesia, pruvitis) and 6 clinical signs (pretibial edema, skin induration, hyperpigmentation, redness, venous ectasia, pain on calf compression) associated with PTS. For each symptom and sign, a score is assigned on a scale of 0 for absent to 3 for severe. These numbers are then summed to give the final Villalta score.^11^ A score of 5 or greater confirms the diagnosis of PTS; a score of 5 to 9 characterizes mild PTS, 10 to 14 moderate PTS, and 15 or greater severe PTS.^11^ This score has also been shown to correlate with patient-perceived quality of life and can be used to assess the effectiveness of treatment.

Following insufficient lysis of the thrombi in our patient’s right and left LE, CDT was pursued. The goal was to reduce the clot burden as much as possible in order to improve long-term outcomes.^12^ Most techniques rely on the use of thrombolytic agents, either through systemic or catheter-directed infusion.^13^ In the CaVenT study, a 14.5% decrease in the incidence of PTS at 24 months in patients with acute iliofemoral DVT who received CDT treatment compared with those who received anticoagulation treatment was observed.^14^ In our patient, CDT in the form of an EKOS ultrasound-accelerated thrombolysis (UAT) catheter (EKOS Corp) was introduced with tPA infusion. The EKOS UAT catheter is a method of CDT that delivers ultrasonic energy to the thrombus while tPA is infused through the catheter. This method of thrombolysis has been shown to provide a 50% reduction of thrombi through standard use in more than 90% of patients.^15^ It is interesting to note that the results of the BERNUTIFUL trial showed that there was no significant difference between EKOS and conventional CDT with regard to vascular patency or PTS occurrence.^16^ However, we decided to pursue UAT as this patient had a history of what appears to be an acute on chronic DVT, and we thought that the addition of the ultrasonic energy would enhance thrombolysis in this specific setting. Following 48 hours of EKOS therapy, moderate clot burden was still seen in specific areas.

Mechanical thrombectomy using the Penumbra Indigo CAT8 system was then chosen, given the added benefit of an increased directional suction power. Mechanical thrombectomy is primarily used in patients at high risk of bleeding with tPA as these techniques do not inherently introduce pharmaceutical agents to thrombi.^17^ The 2 most commonly used devices in interventional mechanical thrombectomy are the AngioJet system (Possis Medical, Minneapolis, MN) and the Trellis device (Bacchus Vascular, Santa Clara, CA).^13^ The latter device was recently taken off the market. All mechanical thrombectomy techniques implement a method of thrombus fragmentation, and usually, a method of evacuation. However, this mechanical technique does come with the risks of valvular and vessel wall injury, as well as PE. The Penumbra Indigo mechanical thrombectomy system (Penumbra, Inc, Alameda, CA) is a new, minimally invasive device that implements continuous suction to evacuate thrombi and emboli from VTE.^18^ The device functions by applying external suction through the catheter to the thrombus and uses an accompanying separator device to make sure the site of evacuation remains unobstructed by the fragmented thrombi. The Indigo system was reported to resolve >70% of thrombi in patients with acute iliofemoral DVT, without the need for postoperative CDT or other endovascular treatments.^19^ The ClotTriever Inari device is the most recent emerging mechanical thrombectomy that is currently being investigated in the CLOUT registry.^20^ This device offers the promise of a single setting clot removal using a 13 Fr sheath and should, in theory, reduce the amount of time patients would spend in the intensive care unit.

Pharmocomechanical catheter-directed thrombolysis (PCDT) techniques combining CDT with mechanical thrombectomy have also been used in the treatment of acute proximal DVT. The results of the ATTRACT trial (n = 692) showed no significant difference between patients who received PCDT treatment and those who received anticoagulation therapy alone with regard to the occurrence of PTS 6 to 24 months following therapy.^21^ The ATTRACT trial, however, included a large number of patients with femoropopliteal DVT, which made the results difficult to apply to patients with more proximal disease. In a subsequent subgroup analysis of patients with DVT involving mainly the deep femoral and iliac veins, PCDT significantly reduced early leg symptoms along with PTS severity scores over 24 months and, more important, reduced the number of patients who developed moderate-or-severe PTS on follow-up resulting in improved quality of life scores.^21^ It should also be noted that 80% of the patients included in the ATTRACT trial had a Villalta score of <15, and almost 50% of them had a Villalta score of <10, which is very different when compared with our patient.^21^

Overall, in patients with symptomatic iliofemoral DVT, available clinical studies support the clinical importance of early thrombus resolution in the prevention of PTS.^3^ On admission, our patient’s PTS was categorized as severe with a Villalta score of 26. Three months following treatment, the patient’s Villalta score decreased by half to 13, recategorizing his PTS as moderate, suggesting that treatment was effective.

## Conclusion

In select cases of patients with proximal iliofemoral DVT and an elevated Villalta score, anticoagulation therapy alone may not be a sufficient management strategy. Multiple interventional treatment options have emerged as safe and effective alternatives and should be considered depending on patients’ specific clinical characteristics.
